# Supplementary figures and images for: Radical genome remodelling accompanied the emergence of a novel host-restricted bacterial pathogen
Source: PLoS Pathog. 2021 May 20;17(5):e1009606. doi: 10.1371/journal.ppat.1009606 (PMC8171923; doi:10.1371/journal.ppat.1009606)

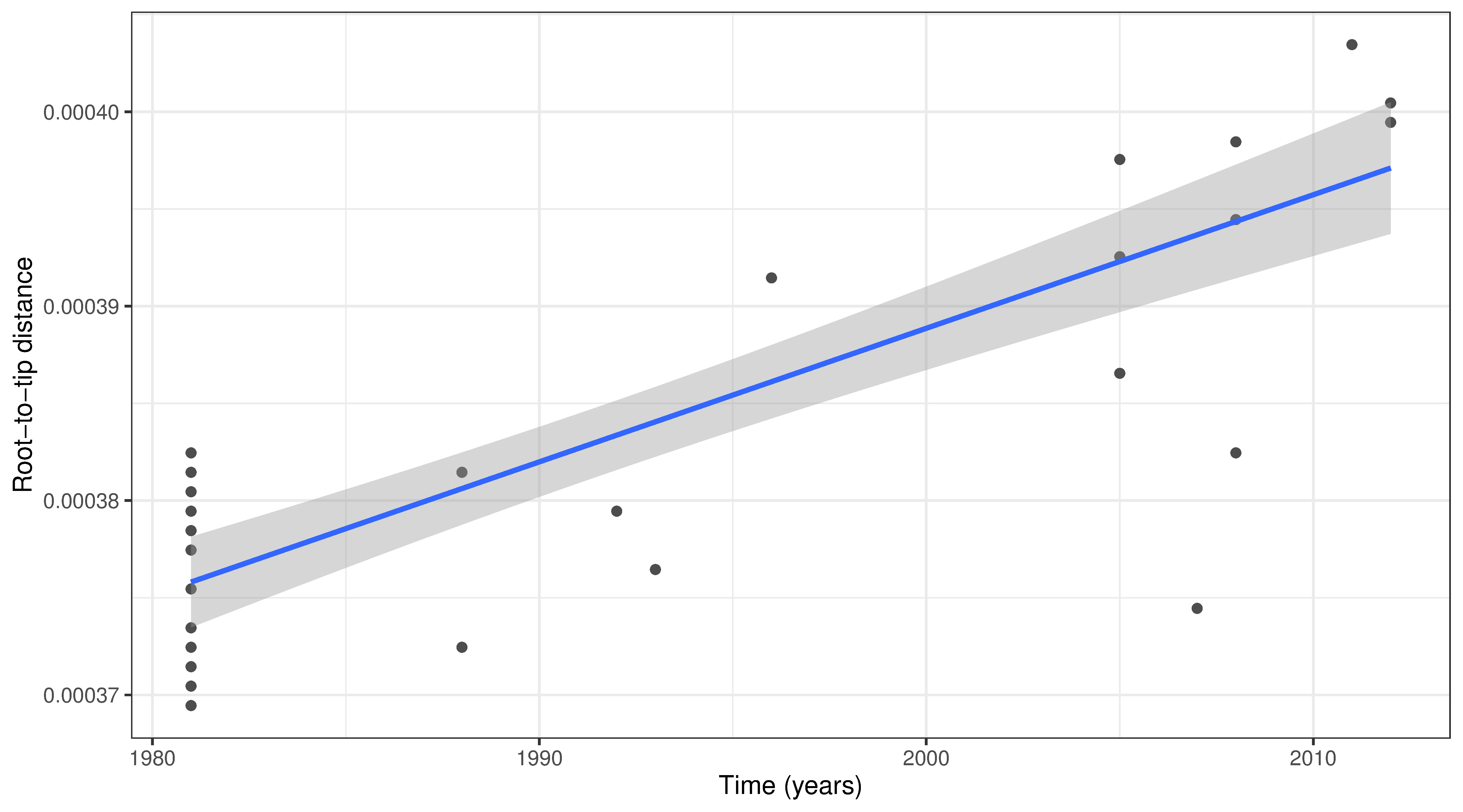

Supplement: S2 Fig — Root-to-tip genetic distance against sampling time estimated from a maximum-likelihood phylogenetic tree built from a core genome alignment of S. aureus subsp. anaerobius sequences. (TIF) [file ppat.1009606.s002.tif]

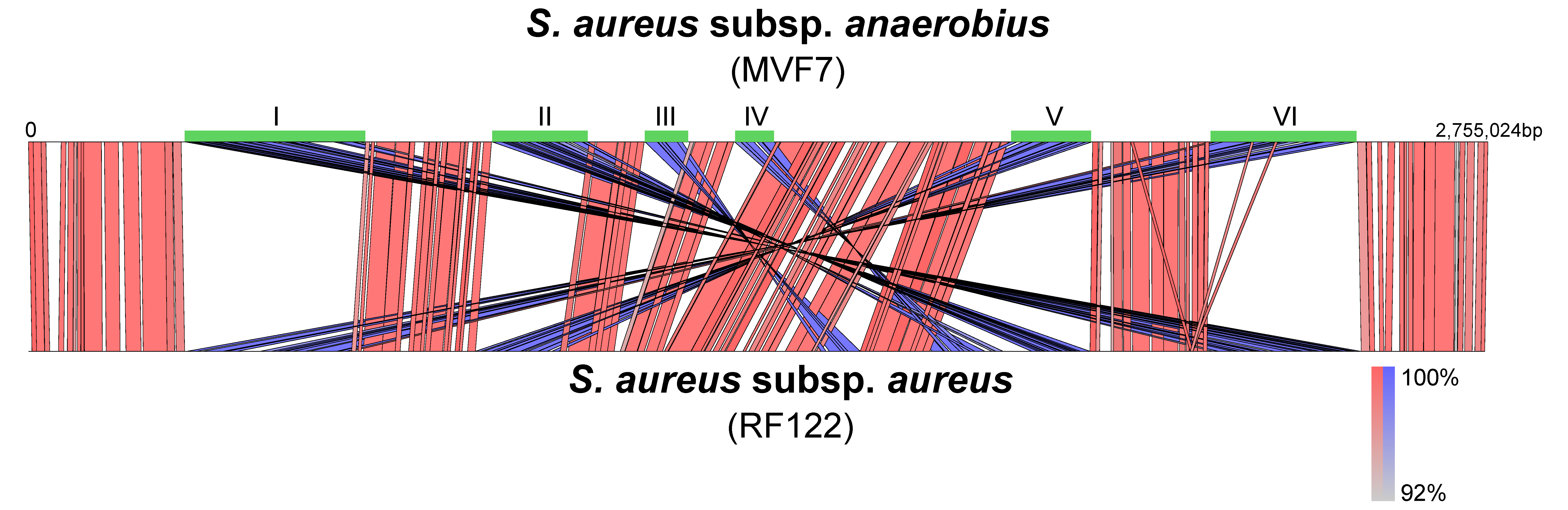

Supplement: S3 Fig — Artemis Comparison Tool (ACT) was used to compare both genomes (MVF7 and RF122, respectively). Red and blue bars indicate regions of similarity in the same and inverted orientation, respectively. The main 6 inverted chromosomal regions are highlighted in green and numbered. (TIF) [file ppat.1009606.s003.tif]

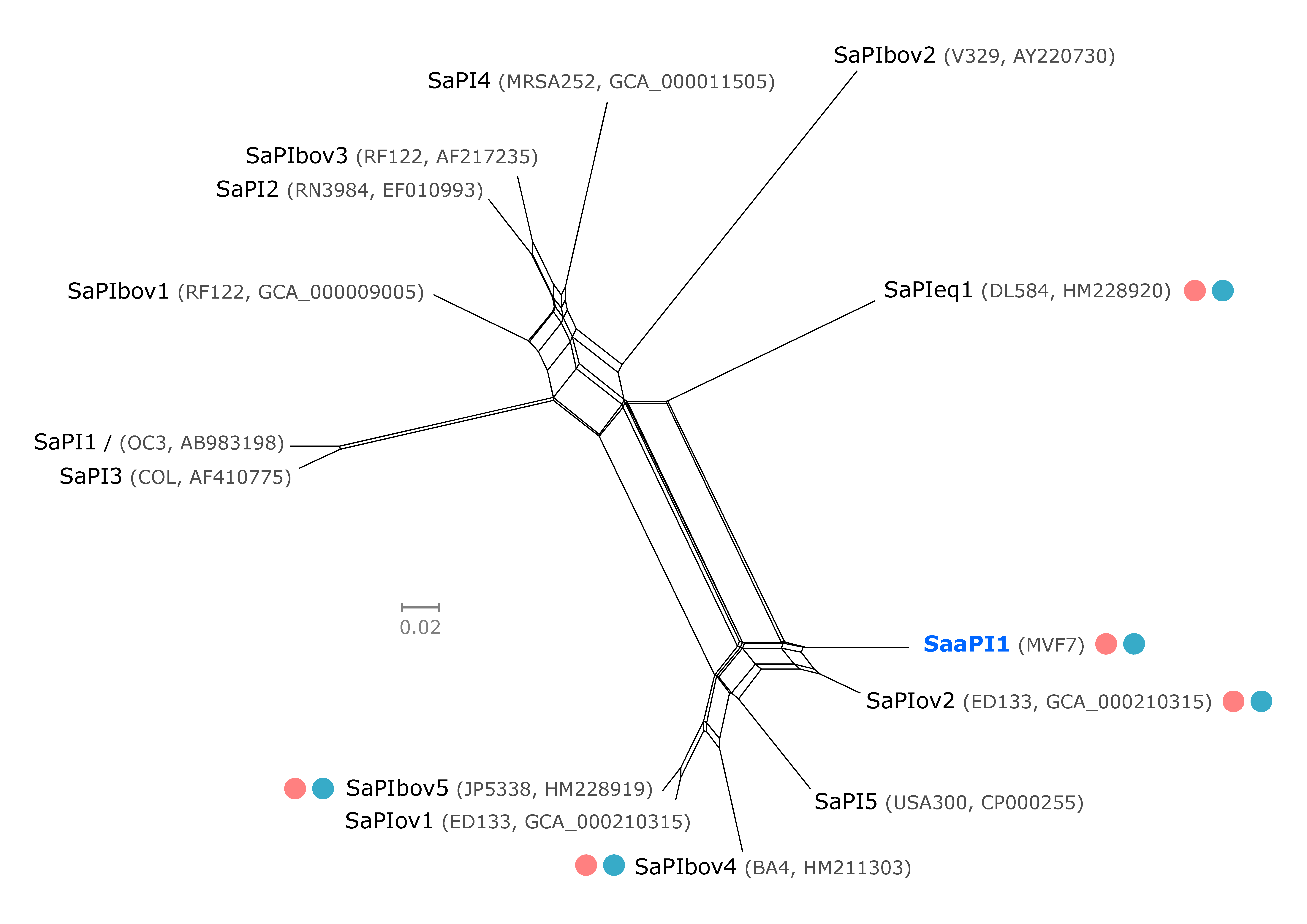

Supplement: S4 Fig — In bold and blue the SaPI found in S. aureus subsp. anaerobius (SaaPIMVF7). The red and blue circles indicate those SaPIs that harbour the genes vwb and scn. Reference sequences are labelled indicating SaPI name, isolate and accession number (of the SaPI sequence when available, of the original genome otherwise). (TIF) [file ppat.1009606.s004.tif]

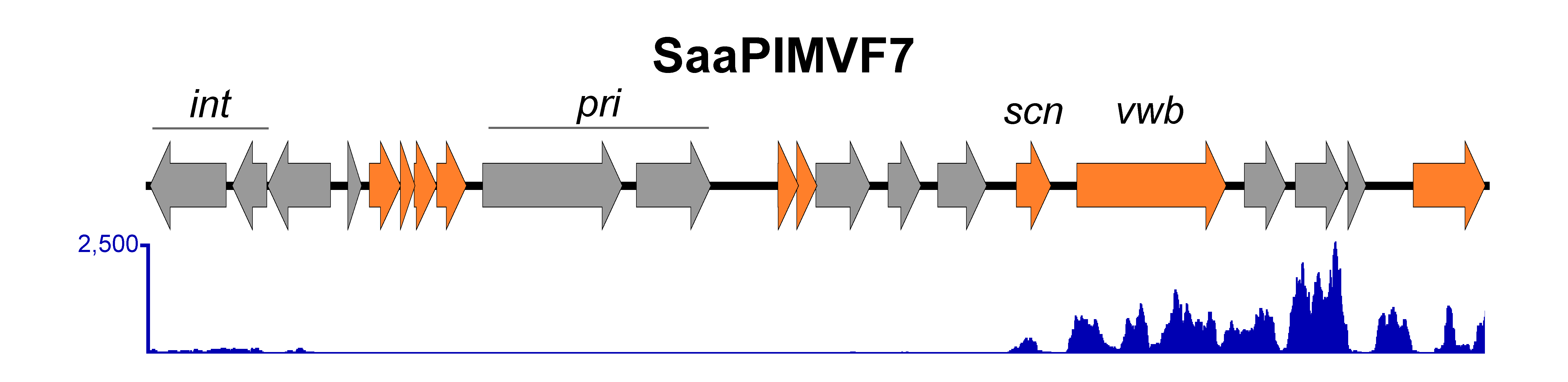

Supplement: S5 Fig — Genes in grey are pseudogenes and genes in orange are intact, according to homology against genes present in previously described S. aureus pathogenicity islands (SaPIs). The histogram in blue represent the genes’ transcription levels (inferred from RNA-seq read coverage). (TIF) [file ppat.1009606.s005.tif]

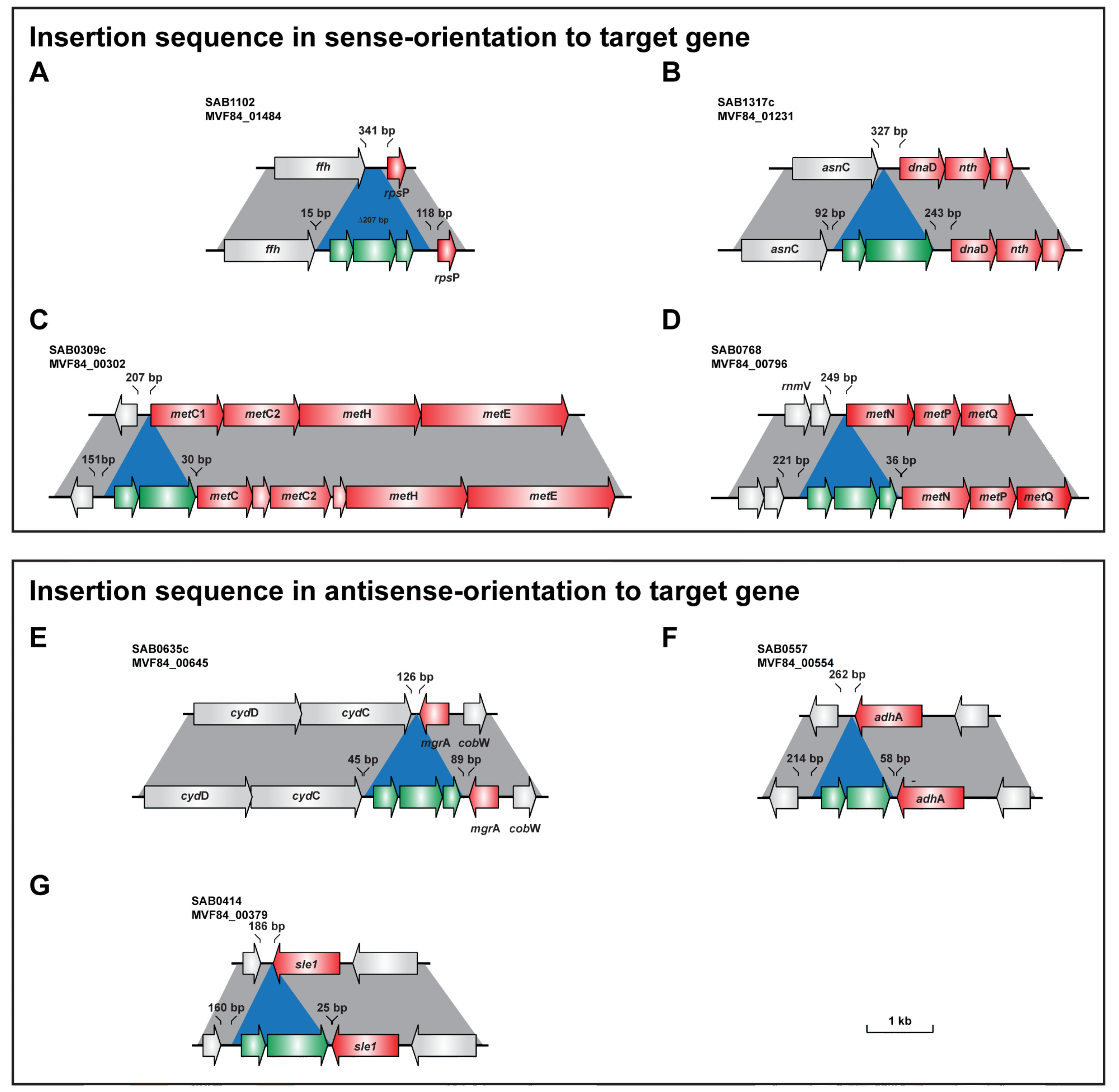

Supplement: S6 Fig — IS loci are shown for S. aureus subsp. anaerobius strain MVF84 and S. aureus strain RF122 representing the ancestral genomic context. (A-D) IS inserted at various distances from the downstream gene start codon. Note that in (A) IS insertion results in a 207 bp deletion in the intergenic region in strain MVF84 relative to strain RF122. (E-G) IS inserted downstream and in antisense orientation of target gene. (E&G) Locus in RF122 shows antisense orientation of downstream gene while in (F) downstream gene is in the same orientation as target gene for IS. (TIF) [file ppat.1009606.s006.tif]

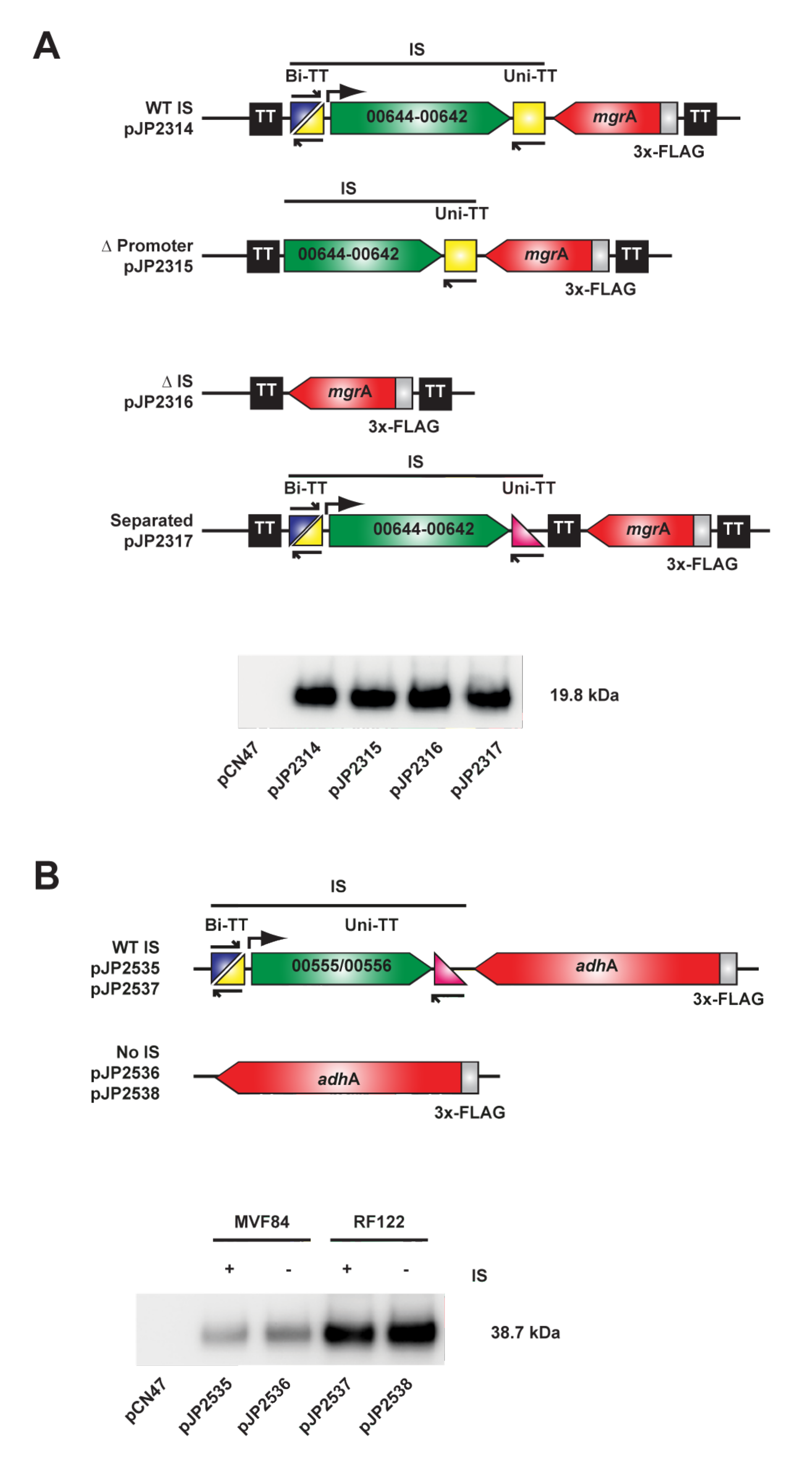

Supplement: S7 Fig — Western blot analysis of the depicted expression constructs for assessing the impact of IS on the expression of (A) MgrA or (B) AdhA from the IS encoded promoter. 3x-FLAG-tagged protein-encoding genes containing or missing the IS were cloned into pCN47 and plasmids introduced into the S. aureus subsp. aureus strain RN4220 Δspa for analysis. For a schematic of the locus in either S. aureus subsp. anaerobius MVF84 or S. aureus subsp. aureus RF122 refer to S4 Fig. (TIF) [file ppat.1009606.s007.tif]

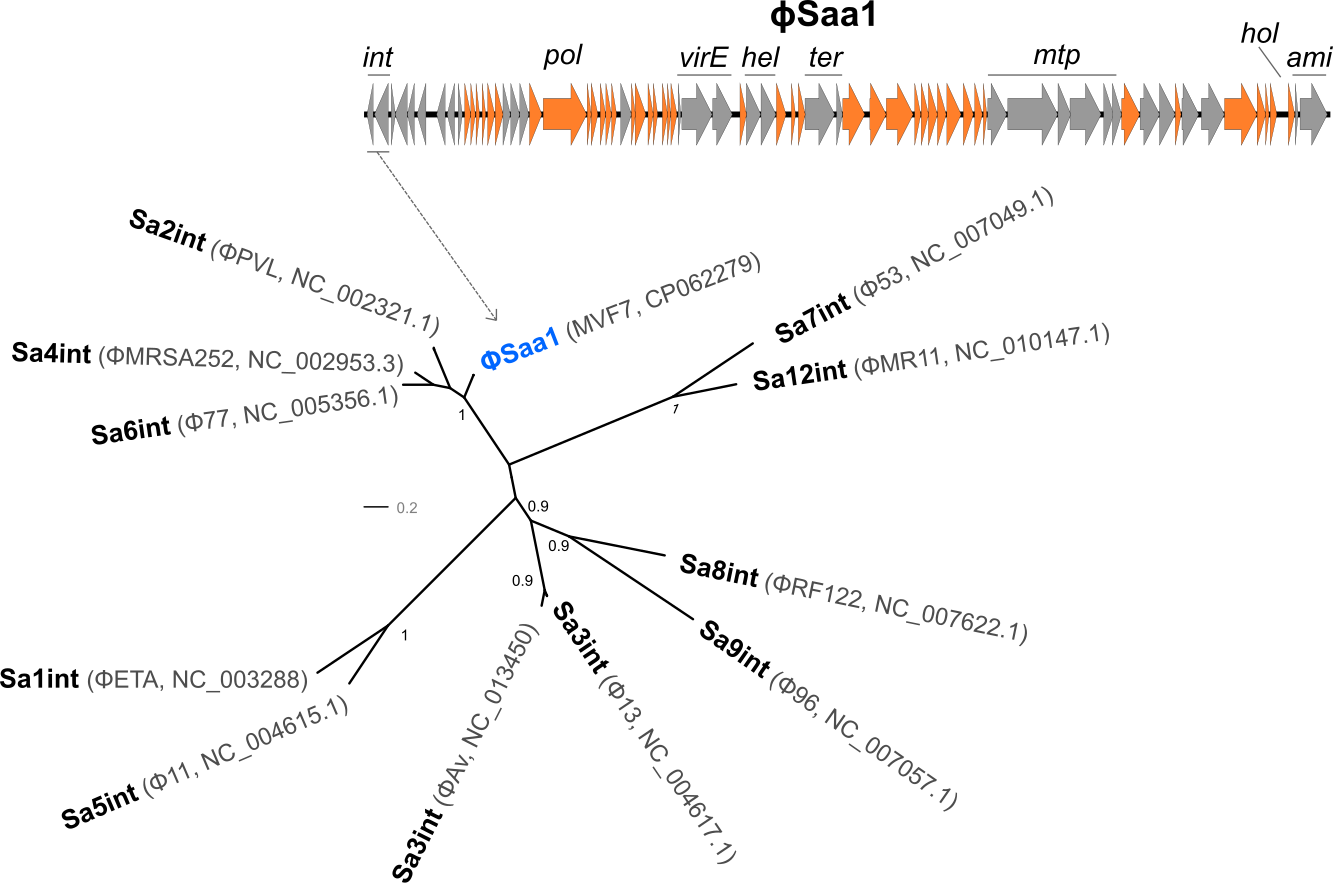

Supplement: S8 Fig — Top: genome map of the phage found in S. aureus subsp. anaerobius (ΦSaa1). Genes in grey are pseudogenes and genes in orange are intact by comparison to Φ2958PVL. Int: integrase; pol: polymerase; virE: virulence protein E; hel: helicase; ter: terminase; mtp: measure tape protein; hol: holin; ami: amidase. Bottom: integrase tree. In bold and blue the integrase of ΦSaa1. Reference sequences are labelled indicating integrase major group, phage and accession number. (TIF) [file ppat.1009606.s008.tif]
